# Short-term anti-proteinuric effect of tacrolimus is not related to preservation of the glomerular filtration rate in IgA nephropathy: A 5-year follow-up study

**DOI:** 10.1371/journal.pone.0188375

**Published:** 2017-11-20

**Authors:** Mi-yeon Yu, Yong-Chul Kim, Ho Suk Koo, Ho Jun Chin

**Affiliations:** 1 Department of Internal Medicine, Seoul National University Hospital, Seoul, Korea; 2 Department of Internal Medicine, Inje University Seoul Paik Hospital, Seoul, Korea; 3 Department of Internal Medicine, Seoul National University Bundang Hospital, Seong-Nam, Korea; 4 Department of Internal Medicine, Seoul National University College of Medicine, Seoul, Korea; 5 Renal Institute, Seoul National University Medical Research Center, Seoul, Korea; Icahn School of Medicine at Mount Sinai, UNITED STATES

## Abstract

**Background:**

The immunosuppressive drug tacrolimus has the short-term effect of reducing proteinuria in patients with immunoglobulin A nephropathy (IgAN). Our study investigated the effects on proteinuria and kidney function after discontinuation of tacrolimus.

**Methods:**

Patients with biopsy-proven IgAN were included in the study and randomly divided into two treatment groups. There was a corresponding control group for each treatment group. The first group included patients treated with tacrolimus (Tac vs non-Tac group) and the second group included patients with a renin angiotensin system blocker (RASi vs non-RASi group). The Tac group received treatment for up to 16 weeks, with the administration of tacrolimus being ceased at the final visit (trial phase). We tracked the patients at 12, 24, 52, and 240 weeks (observational phase). The primary outcomes examined were the percentage change (from the trial phase to the observational phase) of time-averaged proteinuria (TA-proteinuria; g/g creatinine [cr]) and the estimated glomerular filtration rate (eGFR). Time-averaged proteinuria was defined as the average of urine protein to creatinine ratio (UPCR), measured every 3 months during both the trial and observational phases of the study.

**Results:**

A significant reduction in UPCR was observed in the Tac group compared to non-Tac group at the 4 and 8 week visits during the trial phase (*p* = 0.023 and *p* = 0.003, respectively). However, the difference between the Tac group and non-Tac group was not evident in the other review periods, estimated by linear mixed effect model. The percentage change in TA-proteinuria was greater in the Tac group than that in the corresponding control group (116 ± 96% vs. 63 ± 239%, *p* = 0.004). Therefore, during the observational phase, TA-proteinuria was not significantly different between the Tac group and the non-Tac group (1.150 ± 0.733 g/g cr vs. 1.455 ± 2.017 g/g cr, *p* = 0.775). The levels of eGFR throughout the observational phase were not significantly different between the two groups. Furthermore, the mean rate of eGFR change throughout both phases of the study was -6.4 ± 5.9 mL/min/1.73 m^2^/year in the non-Tac group and -5.4 ± 7.9 mL/min/1.73 m^2^/year in the Tac group (*p* = 0.988).

**Conclusion:**

The anti-proteinuric effect of tacrolimus was promptly reversed 3 months after discontinuing the drug. The use of tacrolimus for a short period of time for patients with IgAN temporarily reduces proteinuria, but the data showed no long-term efficacy regarding proteinuria reduction and improvement of renal function.

## Introduction

Proteinuria is a strong predictor of disease progression and kidney survival in patients with immunoglobulin A nephropathy (IgAN) [1–3]. Reich et al. showed that not only proteinuria severity at diagnosis, but also proteinuria exposure over time is an important factor influencing kidney outcomes in IgAN [1]. Furthermore, they demonstrated that kidney outcomes may be improved by proteinuria reduction. Renin-angiotensin-aldosterone system inhibitor (RASi) treatment results in proteinuria reduction and is regarded as one of the main therapies used to treat patients with IgAN and proteinuria [4, 5]. However, RASi also has an antihypertensive effect and therefore has limited use in patients with IgAN and low blood pressure. Consequently, for these patients, a new treatment strategy has been explored.

Tacrolimus has previously been administered to patients with kidney glomerular diseases in an attempt to reduce proteinuria. However, the mechanisms of tacrolimus for reducing proteinuria remains uncertain. Two reports from a study carried out in China stated that up-regulation of the core I B3-Gal-T-specific molecular chaperone gene expression can treat IgAN [6, 7]. Wen et al. demonstrated that tacrolimus restored podocyte damage by stabilizing the expression of the Cabin 1 gene, which is a selective inhibitor of calcineurin [8]. In other studies, tacrolimus has been shown to reduce angiopoietin-like-4 in podocytes, and thereby reduce proteinuria [9, 10]. Although we cannot determine the precise mechanism for proteinuria reduction, it seems likely that tacrolimus is an effective treatment for proteinuria in patients with IgAN. A study by Loeffler et al. revealed that 2 patients with IgAN, who remained proteinuric despite trials of steroid and cyclosporine, responded with cessation of proteinuria after tacrolimus treatment [11]. In another report, tacrolimus induced a rapid proteinuria remission in 14 patients with refractory IgAN [12]. Likewise, we also reported that tacrolimus reduced proteinuria in patients with IgAN and low blood pressure in previous study [13]. Hence, these studies have demonstrated that tacrolimus is an effective short-term method for controlling proteinuria in patients with IgAN.

However, it is not certain whether proteinuria reduction persists after tacrolimus cessation. In this study, we investigated the changes in proteinuria and kidney function after discontinuation of tacrolimus.

## Materials and methods

### Participants

**Fig 1:**
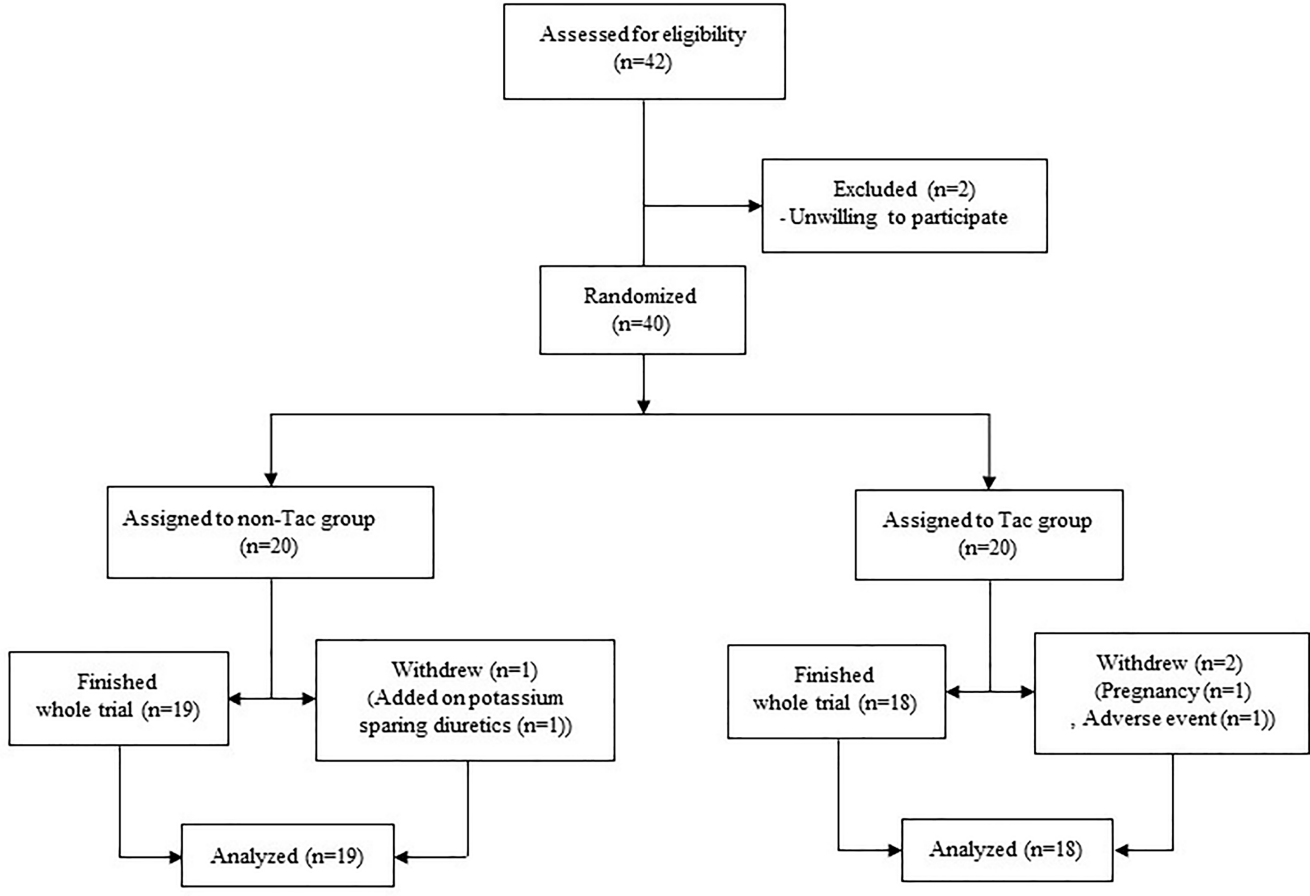
Study algorithm. One patient in the non-Tac group withdrew at the 8-week visit because of the addition of a prohibited drug in another department, one patient in the Tac group withdrew at day 1 after enrollment because of pregnancy and had taken only 2 mg of tacrolimus, and another patient in the Tac group withdrew at the 4-week visit because of general weakness and myalgia related to medication.

The data used in this study were from a previous double-blind randomized controlled clinical trial to verify the anti-proteinuric effect of tacrolimus in patients with IgAN (clinicaltrial.gov identifier: NCT01224028). The study protocol was described in a previous report [13]. Briefly, adult patients with renal biopsy proven IgAN were enrolled and randomized to either the group taking tacrolimus (Tac group) or the placebo group (non-Tac group), with stratification according to the use of a renin-angiotensin-aldosterone inhibitor (RASi vs non-RASi group) where the dosage was not changed throughout a 16-week trial period (Fig 1). The inclusion criteria were a biopsy proven IgAN, age ≥ 18 and < 70 years, serum creatinine (cr) ≤ 1.5 mg/dL or estimated glomerular filtration rate (eGFR) ≥ 45 mL/min/1.73 m^2^, urine albumin to creatinine ratio (UACR) ≥ 0.3 and < 3.0 g/g cr, and blood pressure (BP) less than 130/80 mmHg during the 3-month period before randomization. The eGFR was estimated using the 2009 CKD-EPI cr equation and the IDMS-traceable cr measurement [14]. The initial dose of tacrolimus was 0.1 mg/kg/day administered orally and was titrated to maintain trough levels at 5–10 ng/mL at each visit after randomization. After 8 weeks of randomization, we reduced the dose of tacrolimus to 0.05 mg/kg/day or to half of the dose required to maintain the trough level to 5–10 ng/mL at the 8-week visit and continued this until 16 weeks after randomization. It was planned that tacrolimus would be ceased at the final visit of the trial phase. Of the 40 patients enrolled in the study, we followed 37 who completed the 16-week trial.

### Ethics statement

The study protocol complied with the Declaration of Helsinki and received full approval from the Seoul National University Hospital’s institutional review board (IRB number: H-1002-032-309), which waived the need for informed consent since the study did not infringe on patient privacy or health status.

### Observational phase

All patients were periodically assessed within our hospital. We gathered all clinical parameters regarding medication, urine protein to creatinine ratio (UPCR), UACR, and serum cr from the hospital database of electronic medical records [13]. During the observational period, patients undertook regular laboratory tests including UPCR and serum cr every 2.4 ± 1.0 months (range: 1.1–5.9) and 2.9 ± 1.4 months (range: 1.5–8.8), respectively. We analyzed the data for UPCR and serum cr at 12-weeks (37 patients), 24-week (35 patients), 52-weeks (37 patients), and at the last visit (37 patients), until October 2016, during the observational phase. We accepted the data within 2 weeks of a given follow-up period.

### Outcomes

Time-averaged proteinuria was defined as the average of UPCR, measured every 3 months during both the trial and observational phases of the study. Furthermore, we calculated the percentage change in TA-proteinuria between the trial and observational phases of the study. We calculated the mean rate of eGFR change at each follow-up during the observational phase as compared to the initial level of eGFR at trial phase. A rapid decline in eGFR was defined as a change of ≥ 5 mL/min/1.73 m^2^/year detected during the observational phase. The remission of proteinuria was defined as a UPCR reading of < 0.2 g/g cr at least twice with ≥ 1 month interval. The composite outcome of renal dysfunction was defined as an increase in serum cr level noted during the observational phase whereby there was a ≥ 50% change from baseline (trial phase) or deterioration of renal function to end stage renal disease.

### Statistical analysis

All statistical analyses were performed using SPSS version 22 (IBM, Chicago, Illinois, United States). We used the Mann-Whitney test for analysis of continuous variables and Pearson’s Chi-square test or Fisher’s exact test for qualitative variables. The data throughout the follow-up period was analyzed with linear mixed effect model. To define independent factors related to rapid decline of eGFR, we used multiple logistic regression analysis adjusted with traditional risk factors such as blood pressures, eGFR and UPCR at the initial examination of the trial phase, and TA-proteinuria during the observational phase, and follow-up duration. We used the Cox's proportional hazard model to define independent risk factors for the composite outcome of renal dysfunction adjusted with traditional risk factors. A p-value < 0.05 was used as a criterion of significant difference.

## Results

### Clinical characteristics of patients during the observational period

We enrolled 37 out of 40 patients who had completed the trial to evaluate the anti-proteinuric effect of tacrolimus in patients with IgAN [13]. The characteristics assessed at the final examination in the trial phase are shown in Table 1. The Tac group, who had taken tacrolimus for 16 weeks, showed no differences compared to the corresponding control group, except in relation to UPCR and UACR. Urine protein to creatinine ratio and UACR were lower in the Tac group, 0.649 ± 0.452 vs 0.998 ± 0.479 g/g cr (p = 0.031) and 0.466 ± 0.406 vs 0.711 ± 0.393 g/g cr (p = 0.036), respectively. When the patients were grouped according to the use of a RASi medication at enrollment, the RASi group had also shown no significant differences compared to the non-RASi group, except in the frequency of hypertension (72.2% vs 15.8%, p = 0.001).

**Table 1:** Clinical characteristics of patients at the final visit for clinical study.

	Tacrolimus group			RASi group		
	Non-Tac	Tac	p-value	Non-RASi	RASi	p-value
Number	19	18		19	18	
Age (years)	41.0 ± 12.6	36.8 ± 11.3	0.258	37.0 ± 12.0	41.0 ± 12.0	0.284
Gender (male)	5 (26.3%)	6 (33.3%)	0.641	5 (26.3%)	6 (33.3%)	0.641
SBP (mmHg)	120 ± 11	123 ± 9	0.480	120 ± 11	123 ± 8	0.358
DBP (mmHg)	74 ± 8	74 ± 5	0.663	74.0 ± 8.0	74.0 ± 6.0	0.988
Hypertension	7 (36.8%)	9 (50.0%)	0.419	3 (15.8%)	13 (72.2%)	0.001
Diabetes mellitus	0 (0.0%)	1 (5.6%)	0.486	0 (0.0%)	1 (5.6%)	0.486
UPCR (g/g cr)	0.998 ± 0.479	0.649 ± 0.452	0.031	0.823 ± 0.453	0.824 ± 0.453	0.940
TA-proteinuria (g/g cr)	1.408 ± 0.441	0.611 ± 0.408	0.002	0.816 ± 0.477	0.856 ± 0.484	0.753
UACR (g/g cr)	0.711 ± 0.393	0.466 ± 0.406	0.036	0.594 ± 0.384	0.591 ± 0.453	0.799
Creatinine (mg/dL)	0.99 ± 0.27	1.10 ± 0.32	0.313	0.97 ± 0.24	1.12 ± 0.34	0.245
eGFR (mLmin/1.73 m^2^)	83.1 ± 24.1	77.4 ± 22.9	0.538	86.3 ± 23.9	74.1 ± 21.7	0.118
Albumin (g/dL)	4.2 ± 0.3	4.3 ± 0.3	0.425	4.1 ± 0.3	4.3 ± 0.3	0.061
Protein (g/dL)	7.1 ± 0.5	7.2 ± 0.5	0.988	7.1 ± 0.5	7.2 ± 0.5	0.343
CRP (mg/dL)	0.27 ± 0.69	0.13 ± 0.26	0.098	0.09 ± 0.11	0.32 ± 0.74	0.558
Hemoglobin (g/dL)	13.3 ± 1.3	12.9 ± 1.7	0.425	12.9 ± 1.6	13.2 ± 1.4	0.425
Hematuria	17 (89.5%)	15 (83.3%)	0.660	18 (94.7%)	14 (77.8%)	0.180
RASi	8 (42.1%)	10 (55.6%)	0.413	(-)	(-)	(-)
Tacrolimus	(-)	(-)	(-)	8 (42.1%)	10 (55.6%)	0.413

Values expressed as either mean ± standard deviation, or number (percentage).

Tac: Tacrolimus, RASi: renin-angiotensin-aldosterone system inhibitor, SBP: systolic blood pressure, DB: diastolic blood pressure, cr: creatinine, UPCR: urine protein to creatinine ratio, TA-proteinuria: time averaged proteinuria calculated as the mean urine protein to creatinine ratio, measured every 3 months during both the trial phase of the study, UACR: urine albumin to creatinine ratio, eGFR: estimated glomerular filtration rate by the equation of CKD-EPI, CRP: C-reactive protein, Hematuria: defined as RBC ≥5 under 400-fold magnification examined by light microscopy. P-values were estimated using the Mann-Whitney test.

The patients were followed up for an average of 57.9 ± 13.8 months (range: 22.8–66.7), until October 2016. Among the patients in the Tac group, tacrolimus was stopped at the last visit of the trial phase in 13/18 patients, within 15 days after the last visit in 4/18 patients, and at 15.8 months after the last visit in 1/18 patients who wanted to use it for a longer period. During the observational phase, serum uric acid lowering medication was prescribed more frequently in the Tac group compared to the non-Tac group (33.3% vs 5.3%, p = 0.042) (Table 2). Anti-hypertensive medication other than RASi was used more frequently in the RASi group compared to the non-RASi group (77.8% vs 31.6%, p = 0.008). Additionally, high dose steroid therapy was administered to 47.4% of patients in the non-RASi group and 11.1% of patients in the RASi group (p = 0.029).

**Table 2:** Medication during observation period after cessation of the clinical study.

	Tacrolimus group			RASi group		
	Non-Tac	Tac	p-value	Non-RASi	RASi	p-value
Number	19	18		19	18	
Aspirin (%)	15.8	27.8	0.447	5.3	38.9	0.019
Oral hyperglycemic agent (%)	10.5	10.1	1.000	5.3	16.7	0.340
Anti-hypertensive drug (%)	47.4	61.1	0.402	31.6	77.8	0.008
Prednisolone (%)	31.6	27.8	0.800	47.4	11.1	0.029
Prednisolone with cyclophosphamide (%)	5.3	0.0	1.000	5.3	0.0	1.000
Tacrolimus (%)	0.0	11.1	0.230	10.5	0.0	0.486
Omega-3 Fatty acid (%)	36.8	22.2	0.476	42.1	16.7	0.151
Statin (%)	52.6	44.4	0.618	42.1	55.6	0.413
Allopurinol or febuxostat (%)	5.3	33.3	0.042	15.8	22.2	0.693

Tac: Tacrolimus, RASi: renin-angiotensin-aldosterone system inhibitor, Anti-hypertensive agent: Medication to control hypertension other than RASi, Prednisolone: started with dosage of 1 mg/kg/day prednisolone, cyclophosphamide: started with dosage of 2 mg/kg/day oral cyclophosphamide, Tacrolimus: continued tacrolimus medication after finishing the clinical study in 1 patient and restarted tacrolimus medication in 1 patient after cessation of the clinical study, Omega-3 fatty acid: medication of Omacor®.

### Proteinuria and eGFR during observational phase in Tac group

**Fig 2:**
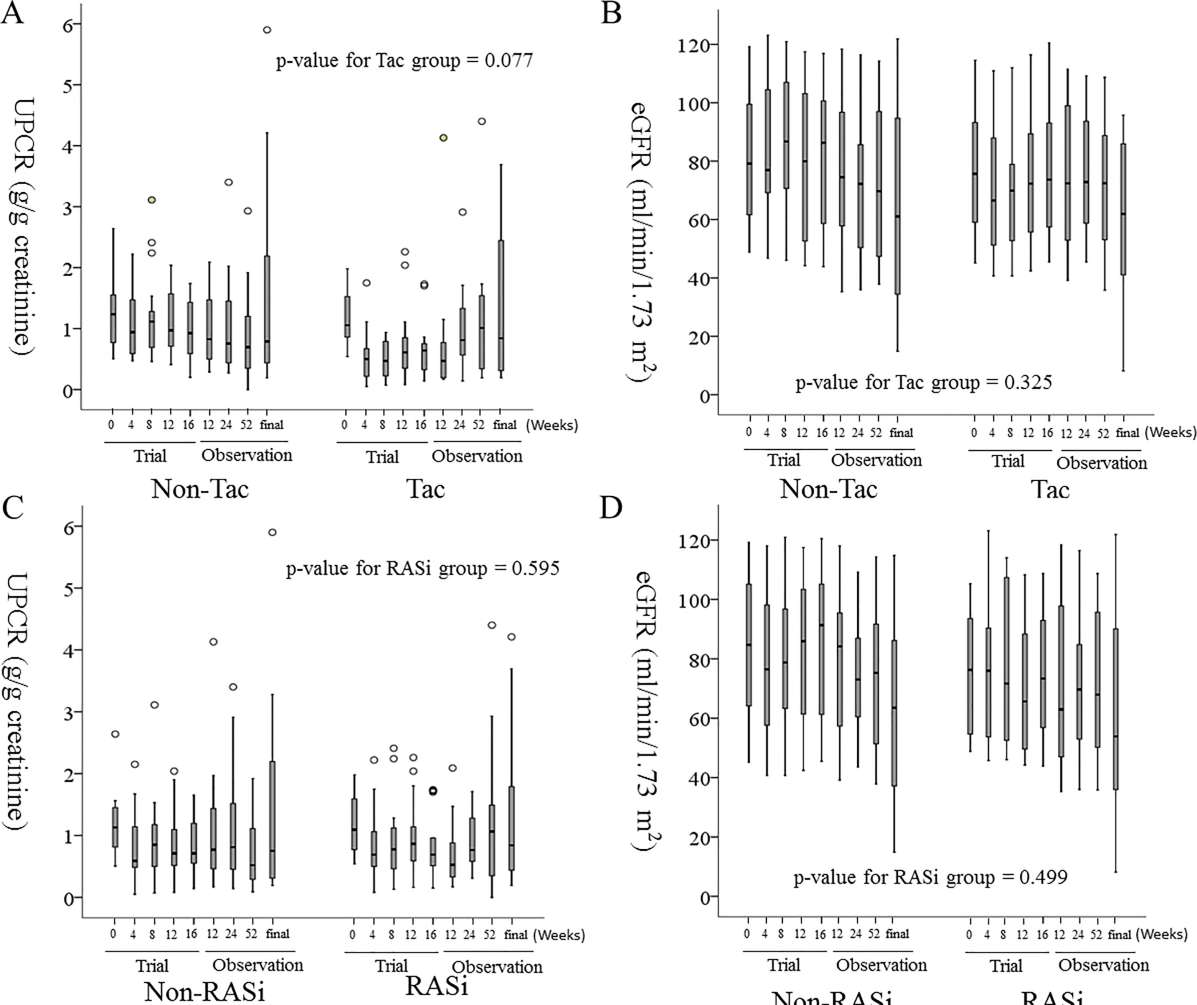
The changes in UPCR (A, C) and eGFR (B, D) during the follow-up period after cessation of the clinical study. From left to right, each box-plot represents the follow-up periods of 0-weeks, 4-weeks, 8-weeks, 12-weeks and 16-weeks of the trial phase, and 12-weeks, 24-weeks, 52-weeks, and the final visit by October 2016 of the observational phase. A. The p-value for tests of between two groups was 0.130. B. The p-value for tests of between-subjects effects was 0.543. C. The p-value for tests of between-subjects effects was 0.830. D. The p-value for tests of between-subjects effects was 0.488. Tac: Tacrolimus, RASi: renin-angiotensin-aldosterone system inhibitor, UPCR: urine protein to creatinine ratio, eGFR: estimated glomerular filtration rate by the equation of CKD-EPI. The p-value estimated by linear mixed effect model.

The UPCR was lower in the Tac group compared to the corresponding control group at the 4-week and 8-week visits during the trial phase (p = 0.023 and p = 0.003, respectively). However, this difference between the Tac and non-Tac group was not evident at the other follow-up periods, estimated by linear mixed effect model (Fig 2). The UPCR at 12, 24, and 52-weeks, and at the final follow-up during the observational phase were 0.970 ± 0.587 g/g cr, 0.841 ± 0.503 g/g cr, 1.222 ± 1.160 g/g cr, and 1.611 ± 1.410 g/g cr at in the Tac group, and 0.783 ± 0.675 g/g cr, 0.908 ± 0.466 g/g cr, and 1.011 ± 0.779 g/g cr, and 1.033 ± 0.726 g/g cr in the corresponding control group, respectively. The TA-proteinuria during the observational phase was 1.455 ± 2.017 g/g cr in the non-Tac group and 1.150 ± 0.733 g/g cr in the Tac group Moreover, there was no significant difference between the two groups (p = 0.775). The mean percent change in TA-proteinuria between the trial and observational phases was higher in the Tac group compared to the non-Tac group (116 ± 96% vs 63 ± 239%, p = 0.004). In the Tac group, UPCR at 4-, 8-, 12-, and 16-weeks was lower compared to that observed at baseline in the trial phase. However, UPCR at 12-, 24-, and 52-weeks, and at the final follow-up during the observational phase were not significantly different from the baseline level observed during the trial phase. The levels of eGFR throughout the observational phase were not significantly different between the Tac and non-Tac groups (Fig 2). Furthermore, the mean rate of eGFR change across both phases of the study was -6.4 ± 5.9 mL/min/1.73 m^2^/year in the non-Tac group and -5.4 ± 7.9 mL/min/1.73 m^2^/year in the Tac group (p = 0.988).

### Proteinuria and eGFR during the observational phase in the RASi group

The UPCRs were not significantly different between the RASi group and the corresponding control group throughout both the trial and observational phases (Fig 2). During the observational phase, TA-proteinuria was 1.488 ± 2.024 g/g cr in the non-RASi group and 1.115 ± 0.694 g/g cr in the RASi group, and there was no significant difference between the two groups (p = 0.916). The mean percentage change in TA-proteinuria between the trial and observational phases was not significantly different between the RASi and non-RASi groups (59 ± 96% vs 117 ± 239%, respectively, p = 0.663). Likewise, the levels of eGFR throughout the observational phase were not significantly different between the RASi and non-RASi groups (Fig 2). However, the mean rate of eGFR change throughout both phases was -8.7 mL/min/1.73 m^2^/year in the non-RASi group and -3.0 mL/min/1.73 m^2^/year in the RASi group (p = 0.022).

### Outcomes of proteinuria and eGFR

**Fig 3:**
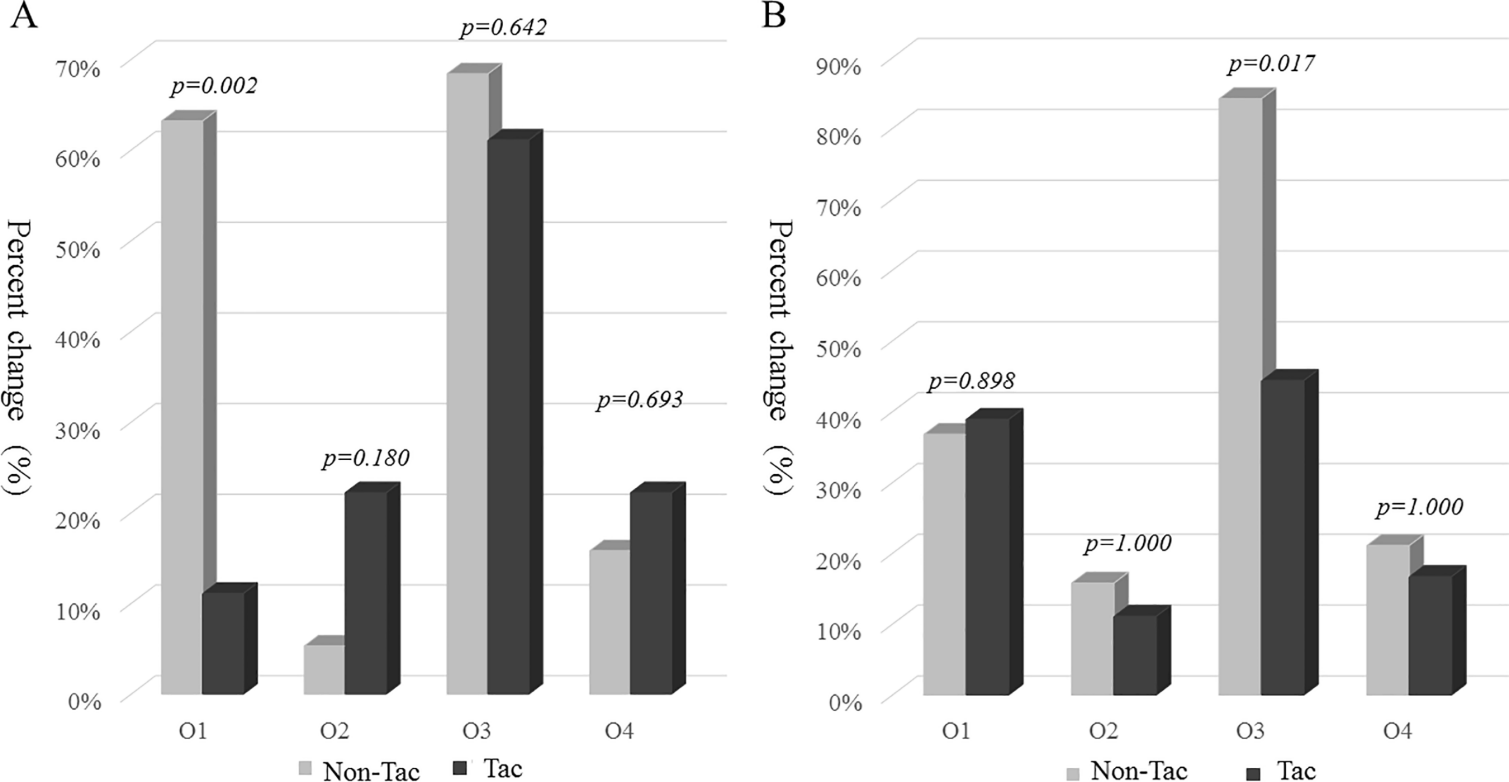
The difference in outcome parameters between groups. Outcome parameters represent the followings; O1, decrease of time-averaged proteinuria from trial phase and to the observational phase; O2, remission of UPCR <0.2 g/g cr during observational phase; O3, rapid decline of eGFR ≥5 mL/min/1.73 m^2^ during observational phase; O4, composite outcome of increase in serum cr level (≥50% from baseline) noted during observational phase or deterioration of renal function to end stage renal disease. Outcome parameters were compared by Chi-square test or Fisher’s exact test according to the number of each cell in the tacrolimus group (A) and RASi group (B).

We compared four outcomes between the groups (Fig 3). The non-Tac group showed a higher rate of a decreased TA-proteinuria in the observational phase compared to the trial phase compared to Tac group (63.2% vs 11.1%, p = 0.002). Additionally, the RASi group showed lesser probability of rapid decline of eGFR compared to the non-RASi group (44.4% vs 84.2%, respectively, p = 0.017). The RASi group had a 0.150-fold risk reduction (95% CI of relative risk: 0.032–0.703, p = 0.016) for rapid decline in eGFR compared to the non-RASi group by multiple logistic regression analyses, adjusted with conventional risk factors for renal functional progression in IgAN, such as SBP, DBP, eGFR and UPCR at the initial examination of the trial phase, time-averaged proteinuria during follow-up period, and follow-up duration. Conversely, the Tac group was not related to risk reduction (Table 3). The independent risk factors for the composite outcome of renal dysfunction were the mean rate of eGFR change throughout both phases, and the initial eGFR level at trial phase, analyzed by Cox’s proportional hazard model adjusted with conventional risk factors such as blood pressure, UPCR, and eGFR at baseline, TA-proteinuria during the follow-up period, a pathologic parameter of endocapillary hypercellularity by the Oxford’s classification, and duration of follow-up.

**Table 3:** The relationship between groups and rapid decline of eGFR during follow-up period.

	B	RR (95% CI)		Wald	p-value
RASi group (RASi)	-1.897	0.150	0.032–0.703	5.797	0.016
Tacrolimus group (Tac)	uc	uc	uc	uc	0.890

Multiple logistic regression analysis was done for the incidence of rapid decline of eGFR (≥5 mL/min/1.72 m^2^/year) during the observational phase, adjusted with SBP, DBP, eGFR and UPCR at the initial examination of the trial phase, time-averaged proteinuria during follow-up period, and follow-up duration. uc: uncountable.

## Discussion

In our previous study, tacrolimus had an anti-proteinuric effect during 16 weeks of treatment (trial phase) of patients with IgAN patients and mild to moderate proteinuria [13]. After tacrolimus was discontinued at the final visit of the trial phase, TA-proteinuria and eGFR were not significantly different between the Tac and non-Tac groups during the 5-year follow-up (observational phase). Additionally, we found persistent use of a RASi, which was used during the trial and observational phases, was related to a reduced risk of rapid decline in eGFR in patients with IgAN.

Tacrolimus was effective in controlling proteinuria in patients with IgAN [11–13]. Recently, two studies conducted meta-analysis of randomized controlled trials (RCTs) and showed that tacrolimus might provide kidney protection in patients with IgAN [15, 16]. Along with the results of these studies, several studies have investigated the mechanisms of tacrolimus in reducing proteinuria. Tacrolimus was considered to have multifocal mechanisms for reducing proteinuria, such as immunosuppressive mechanisms by suppression of the nuclear factor of activated T cell activity in podocytes [17, 18], intraglomerular hemodynamic changes [19–21], inhibition of the vascular permeability factor [22], and a direct effect on podocytes [8–10, 23, 24]. Among these mechanisms, we suggested that the early anti-proteinuria effect of tacrolimus was due to reversible hemodynamic changes rather than to other structural changes. In our study, the 16-week use of tacrolimus decreased UPCR in patients with IgAN compared to those taking the placebo, especially in the first 2 months [13]. At 8 weeks, the UPCR and UACR were lowest, but serum cr was highest. These changes are similar to those seen after using a calcineurin inhibitor (CNI). Tacrolimus is a CNI and its nephrotoxicity is already well known. In acute CNI nephrotoxicity, vasoconstriction of the afferent arterioles causes glomerular permeability to protein to decrease and results in reduction of proteinuria [25]. When tacrolimus level was decreased, subsequently, serum cr returned to within the normal range and UPCR was slightly increased but still lower than the non-Tac group. The lower UPCR in the Tac group compared to the non-Tac group during the trial phase despite the later cr has recovery, is difficult to explain solely by hemodynamic changes. Therefore, the rapid decrease of proteinuria in the early stage may be due to hemodynamic changes, but the overall decrease may be the combination of the various mechanisms mentioned above [16–23]. Time-averaged proteinuria is the most reliable predictor of kidney survival [1]. Despite proteinuria reduction during the trial phase, the use of tacrolimus for 16 weeks did not have a significant effect on TA proteinuria or the rate of eGFR change during the 57.9 ± 13.8 months of follow-up. Although UPCR was lower in the Tac group compared to the non-Tac group during the trial phase, it increased within 3 months after stopping tacrolimus. There was no difference in the total dose of proteinuria during the trial and observational period between the two groups. Therefore, we suggested that the total amount of proteinuria may have a stronger impact on the kidney function than the exposure period. However, two aspects need to be considered before asserting that tacrolimus has no long duration effect. The first aspect is tacrolimus dosage. Several studies evaluating the direct effects of tacrolimus on podocytes have shown that structural changes occur in podocytes when tacrolimus is administered, hence suggesting long duration effects of tacrolimus [8, 23, 24]. In these aforementioned studies, a tacrolimus dose of approximately 0.2–1 mg/kg/day was given; however, these particular studies were performed on animals. In our study, the initial dose of tacrolimus was 0.1 mg/kg/day and was reduced to 0.05 mg/kg/day [13]. Similarly, in other human studies, tacrolimus was administered at 0.05–0.1 mg/kg/day in patients with IgAN to control proteinuria [12, 26]. Therefore, the short-term anti-proteinuric effect rather than a long duration effect, can only be considered as we did not use a sufficient dose of tacrolimus to cause structural change. The second aspect requiring consideration is that we enrolled patients with mild IgAN. The mean UPCR and UACR were 1398 ± 809 and 1098 ± 635 mg/g cr, respectively. These values were lower than the baseline values in other studies [12, 26]. The mean eGFR was 79.6 ± 21.6 mL/min/1.73 m^2^. In these patients, podocyte injury may be mild, and therefore the effect of tacrolimus induced kidney protection may not be obvious.

In this study, the 3-month treatment did not cause a difference in TA-proteinuria and eGFR between the Tac group and the non-Tac group. Based on several other studies demonstrating the efficacy of tacrolimus, the trial phase would not be a long enough in duration to result in long term effects. However, at present, no guidelines have been established and thus treatment period varies from 3 months to 1 year [12, 13, 26]. Tacrolimus is an immunosuppressant agent that requires attention to dose and duration. High dose and long-term treatment with tacrolimus may induce tubular fibrosis and damage of kidney function [27]. Considering nephrotoxicity, infection due to immunosuppression, and side effects such as gastrointestinal discomfort, abnormal liver function, and high glucose levels, it is important to determine the shortest duration of treatment that can be effective [13, 15, 16, 26, 28]. In addition, it is important to check tacrolimus trough levels through regular visits to hospital and blood tests. When patients should be prescribed tacrolimus over a long period, they tend to find this dissatisfactory and inconvenient due to the additional visits and tests they must undergo. Therefore, further research on the efficacy and mechanism of tacrolimus, as well as developing a consensus on the appropriate dose and duration to obtain therapeutic response is required.

Another study revealed that after cessation of a 6-month tacrolimus treatment, proteinuria relapsed in 4/9 (44.4%) patients [12]. The time range of relapse was from 2 to 5 months, similar to our result. In China, a prospective observational study revealed only 5/14 patients with steroid-resistant nephrotic syndrome experienced relapse, where 3 of the cases occurred during the treatment period and 2 occurred after stopping a 12-month tacrolimus treatment. However, in that study, none of the 2 patients with IgAN showed recurrence of proteinuria [26]. The difference in relapse rates between studies is considered to be due to various factors, such as treatment duration, severity of IgAN, and combined therapy. An explanation for the high recurrence of IgAN after cessation of tacrolimus, is still uncertain. Hence, given that relapse of IgAN is an important prognostic factor and a treatment director, we need more consideration and additional studies are needed.

RASi was the main drug for controlling blood pressure and proteinuria in patients with IgAN [29]. There was no difference in TA-proteinuria and eGFR between the RASi group and its control group. However, we found that persistent use of the RASi, which was permitted to be used during the trial and observational phases, was related to a reduction in the risk of developing a rapid decline of eGFR in patients with IgAN. We propose that extending the follow-up duration or increasing the number of patients included in the study, eGFR will be statistically better in the RASi group than the non-RASi group. Therefore, in patients with mild IgAN who do not have low blood pressure and a risk of hyperkalemia, a RASi may be a suitable treatment. The reason why there was no difference in TA-proteinuria between the two groups might be that the non-RASi group had more patients with prednisolone.

This study was limited by the patient population, which had biopsy confirmed IgAN with mild to moderate proteinuria. Hence, our results not necessarily be generalized to all patients with IgAN. Furthermore, we were not able to confirm pathologic changes through biopsy, after the trial and observational phases. Identifying pathologic changes through additional animal experiments using different drug dose and treatment duration, may be the basis for our results that the tacrolimus will not show a prolong effect.

In conclusion, the anti-proteinuric effect of tacrolimus was promptly reversed 3 months after discontinuation of the drug. The use of tacrolimus for a short period of time for patients with IgAN and mild to moderate proteinuria, temporarily reduces proteinuria, but there seems to be no long-term efficacy.

## Supporting information

S1 File Individual patient information. (XLSX)
