# Correction: Short-term anti-proteinuric effect of tacrolimus is not related to preservation of the glomerular filtration rate in IgA nephropathy: A 5-year follow-up study

**DOI:** 10.1371/journal.pone.0192266

**Published:** 2018-01-29

**Authors:** Mi-yeon Yu, Yong-Chul Kim, Ho Suk Koo, Ho Jun Chin

The x-axis labels for Fig 2D are cut-off. The x-axis labels for Fig 3B are incorrect. Please see the correct Fig 2 and Fig 3 here.

**Fig 2:**
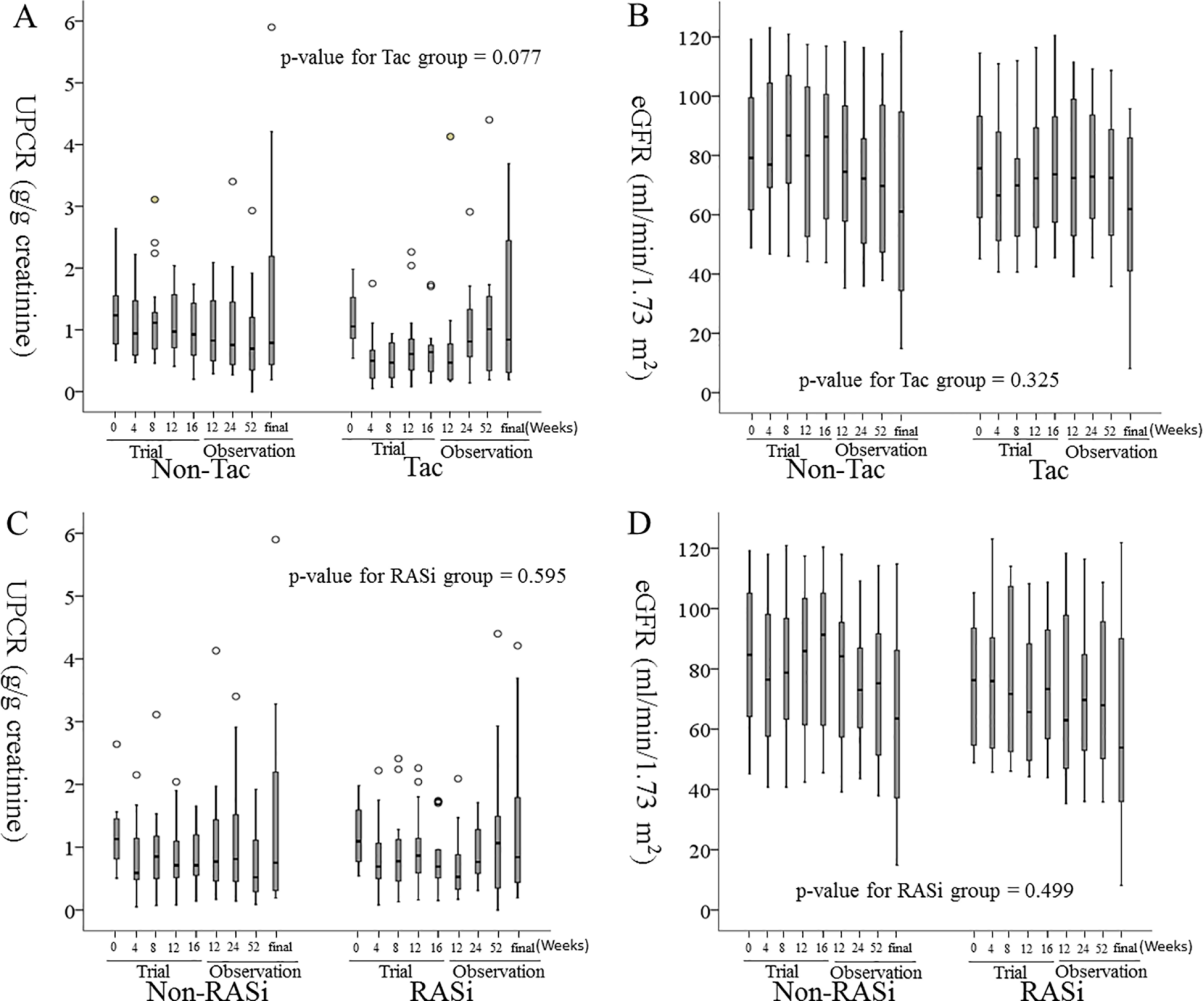
**The changes in UPCR (A, C) and eGFR (B, D) during the follow-up period after cessation of the clinical study.** From left to right, each box-plot represents the follow-up periods of 0-weeks, 4-weeks, 8-weeks, 12-weeks and 16-weeks of the trial phase, and 12-weeks, 24-weeks, 52-weeks, and the final visit by October 2016 of the observational phase. A. The p-value for tests of between two groups was 0.130. B. The p-value for tests of between-subjects effects was 0.543. C. The p-value for tests of between-subjects effects was 0.830. D. The p-value for tests of between-subjects effects was 0.488. Tac: Tacrolimus, RASi: renin-angiotensin-aldosterone system inhibitor, UPCR: urine protein to creatinine ratio, eGFR: estimated glomerular filtration rate by the equation of CKD-EPI. The p-value estimated by linear mixed effect model.

**Fig 3:**
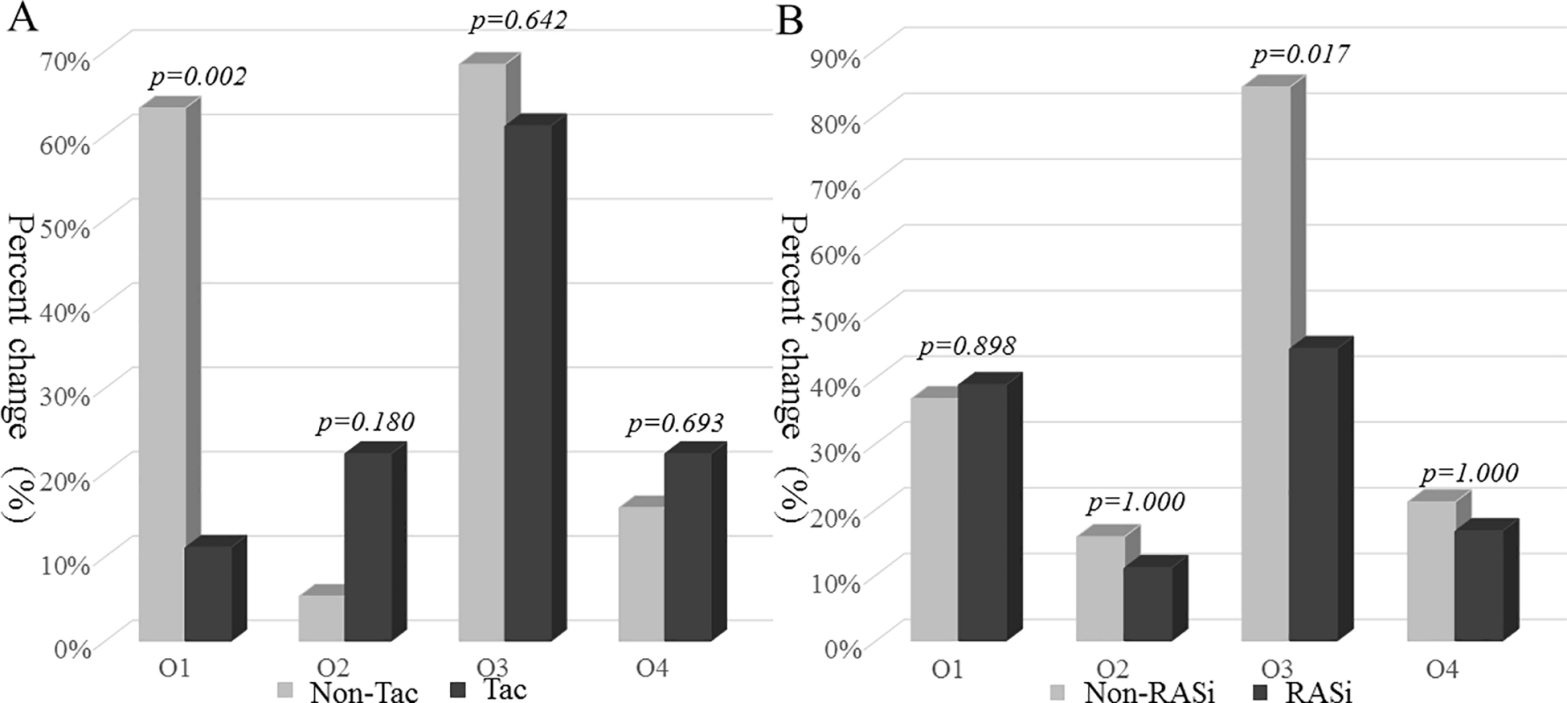
The difference in outcome parameters between groups. Outcome parameters represent the followings; O1, decrease of time-averaged proteinuria from trial phase and to the observational phase; O2, remission of UPCR <0.2 g/g cr during observational phase; O3, rapid decline of eGFR ≥5 mL/min/1.73 m^2^ during observational phase; O4, composite outcome of increase in serum cr level (≥50% from baseline) noted during observational phase or deterioration of renal function to end stage renal disease. Outcome parameters were compared by Chi-square test or Fisher’s exact test according to the number of each cell in the tacrolimus group (A) and RASi group (B).
